# Analysis of vaccine responses after anti-CD20 maintenance in B-cell lymphoma in the Balearic Islands. A single reference center experience

**DOI:** 10.3389/fimmu.2023.1267485

**Published:** 2023-11-01

**Authors:** Antonio Gutierrez, Aser Alonso, Marta Garcia-Recio, Sandra Perez, Lucia Garcia-Maño, Jordi Martinez-Serra, Teresa Ros, Mercedes Garcia-Gasalla, Joana Ferrer, Oliver Vögler, Regina Alemany, Antonio Salar, Antonia Sampol, Leyre Bento

**Affiliations:** ^1^Service of Hematology, University Hospital Son Espases/Health Research Institute of the Balearic Islands (IdISBa), Palma, Spain; ^2^Service of Internal Medicine and Infecious Diseases, University Hospital Son Espases/Health Research Institute of the Balearic Islands (IdISBa), Palma, Spain; ^3^Service of Immunology, University Hospital Son Espases, Palma, Spain; ^4^Group of Advanced Therapies and Biomarkers in Clinical Oncology, Health Research Institute of the Balearic Islands (IdISBa), Research Institute of Health Sciences (IUNICS), University of the Balearic Islands, Palma, Spain; ^5^Group of Clinical and Translational Research, Department of Biology, University of the Balearic Islands, Palma, Spain; ^6^Service of Hematology , Hospital Clinico Universitario Virgen de la Arrixaca, Murcia, Spain

**Keywords:** seroconversion, vaccine failure, B-cell aplasia, SARS/CoV-2, anti-CD20 maintenance

## Abstract

**Introduction:**

The use of maintenance approaches with anti-CD20 monoclonal antibodies has improved the outcomes of B-cell indolent lymphomas but may lead to significant peripheral B-cell depletion. This depletion can potentially hinder the serological response to neoantigens.

**Methods:**

Our objective was to analyze the effect of anti-CD20 maintenance therapy in a reliable model of response to neoantigens: SARS-CoV-2 vaccine responses and the incidence/severity ofCOVID-19 in a reference hospital.

**Results:**

In our series (n=118), the rate of vaccination failures was 31%. Through ROC curve analysis, we determined a cutoff for SARS-CoV-2 vaccine serologic response at 24 months from the last anti-CD20 dose. The risk of severe COVID-19 was notably higher within the first 24months following the last anti-CD20 dose (52%) compared to after this period (just 18%) (p=0.007). In our survival analysis, neither vaccine response nor hypogammaglobulinemia significantly affected OS. While COVID-19 led to a modest mortality rate of 2.5%, this figure was comparable to the OS reported in the general immunocompetent population. However, most patients with hypogammaglobulinemia received intravenous immunoglobulin therapy and all were vaccinated. In conclusion, anti-CD20 maintenance therapy impairs serological responses to SARS-CoV-2 vaccines.

**Discussion:**

We report for the first time that patients during maintenance therapy and up to 24 months after the last anti-CD20 dose are at a higher risk of vaccine failure and more severe cases of COVID-19. Nevertheless, with close monitoring, intravenous immunoglobulin supplementation or proper vaccination, the impact on survival due to the lack of serological response in this high-risk population can be mitigated, allowing for the benefits of anti-CD20 maintenance therapy, even in the presence of hypogammaglobulinemia.

## 1 Introduction

Anti-CD20 monoclonal antibodies, like rituximab, have enhanced the outcomes of B-cell lymphoma patients when incorporated into many standard chemotherapy regimens (1). However, a significant advancement was achieved with the introduction of maintenance approaches. These involve periodic infusions of anti-CD20 monoclonal antibodies every 2, 3 or 6 months, ensuring continuous anti-CD20 activity against the minimal residual disease that remains after an initial debulking immunochemotherapy. The use of anti-CD20 maintenance approaches has improved the outcome in terms of longer progression-free (PFS) or overall survival (OS) in B-cell lymphomas such as follicular or mantle lymphoma, as shown in PRIMA (2, 3), BRIGHT (4) or LYMA (5) trials. However, other anti-CD20 monoclonal antibodies, such as obinutuzumab, showed better efficacy results, being able to rescue rituximab-resistant patients but at the cost of greater toxicity (6, 7).

Although anti-CD20 maintenance is generally well-tolerated, there is still some significant toxicity mainly related to peripheral B-cell depletion. This B-cell aplasia is generally complete during anti-CD20 maintenance and, after the last dose of anti-CD20, B-cell counts may need several months to recover or even remain prolonged or persistent in some individuals (8). This may impair serological response to neoantigens, including SARS-CoV-2 spike glycoprotein within SARS-CoV-2 vaccines (9) and, although this is well described, to the best of our knowledge no specific study has focused on patients receiving anti-CD20 maintenance approaches increasing the risk of prolonged B-cell aplasia. At the same time, the COVID-19 pandemic offered us the opportunity to study the vaccine response to a particular neoantigen, related to SARS-CoV-2 virus. In this study, we aim to analyze the effect of anti-CD20 maintenance on SARS-CoV-2 vaccine responses and COVID-19 incidence and severity in a single reference hospital.

## 2 Materials and methods

### 2.1 Study design

We retrospectively selected from the Pharmacy database of Son Espases University Hospital, those alive patients with B-cell lymphomas treated with anti-CD20 maintenance therapy candidates to be included in the study. Inclusion criteria were having received previous or ongoing frontline anti-CD20 maintenance from January 2003 to August 2022, having received at least one dose of any approved SARS-CoV-2 vaccine by August 2022 and willingness to sign the informed consent. Exclusion criteria included not having received at least one dose of any approved SARS-CoV-2 vaccine by August 2022, anti-CD20 maintenance beyond frontline therapy for B-cell lymphoma, previous administration of anti-SARS/CoV-2 monoclonal antibodies or unwillingness to sign the informed consent. The study was approved by the Balearic Islands ethic committee (L99E19746/2020). Clinical characteristics and outcome were obtained from medical records.

### 2.2 Humoral immunodeficiency, SARS-CoV-2 vaccination, and COVID-19

Relevant clinical data was retrospectively obtained from electronic medical records of Son Espases University hospital. They included staging and prognostic factors in B-cell lymphoma, humoral immune status assessed by the level of serum immunoglobulins and the need of immunoglobulin supplementation. Hypogammaglobulinemia was defined as IgG levels below normal levels in our center (500 mg/dL). For SARS-CoV-2 serologic assessment we used a high-throughput chemiluminescent immunoassay (CLIA) platform. Vaccination response was evaluated as the rate of seroconversion after vaccine administration. Seroconversion was defined as conversion from negative to protective titers of IgG anti-S (>260 AU/mL). Vaccination failure was defined as not achieving protective titers after at least 1 vaccination dose. COVID-19 severity was analyzed using Radiographic Assessment of Lung Edema (RALE) score (10, 11) in those patients available (severe and those requiring ICU admission), as they offers an objective, rapid and widely available tool that can be extremely useful, especially when integrated with other clinical data. However, from a practical point of view COVID-19 severity was classified as asymptomatic, mild, severe, or requiring ICU admission.

### 2.3 Statistical methods

Variables following binomial distributions (i.e.: response rate), were expressed as frequencies and percentages. Comparisons between qualitative variables were done using the Fisher Exact Test or Chi-square. Comparisons between quantitative and qualitative variables were performed through non-parametric tests (U of Mann-Whitney or Kruskal-Wallis). To analyze the moment of recovery of the serological response to SARS-CoV-2 vaccination after the last dose of anti-CD20 maintenance, ROC curves were used. Time to event variables (OS and PFS) were measured from the date of therapy onset and were estimated according to the Kaplan-Meier method. Comparisons between the variables of interest were performed by the log-rank test. All p-values reported were 2-sided, and statistical significance was defined at p < 0·05.

## 3 Results

### 3.1 Characteristics of the patients

**Figure 1:**
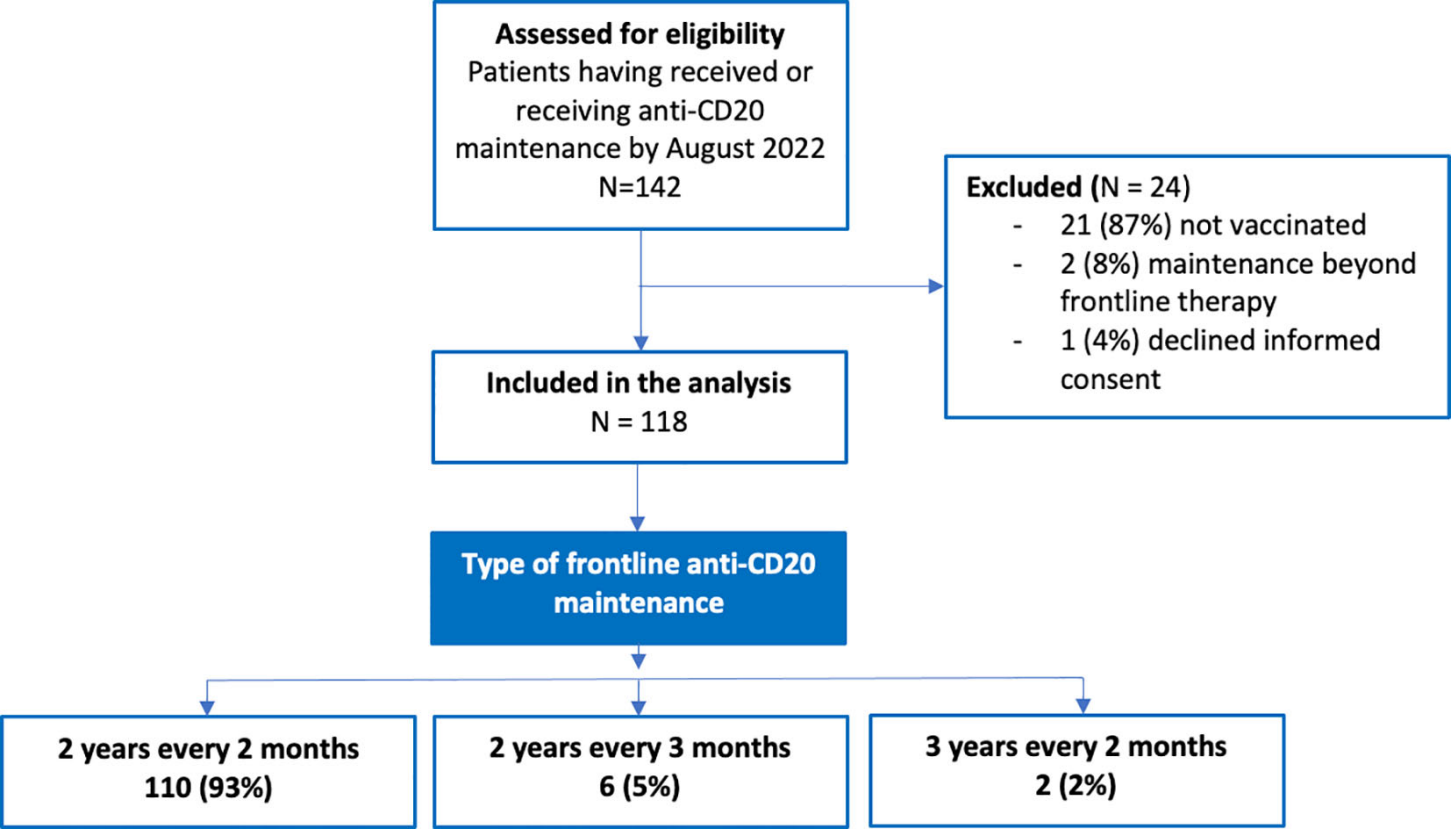
Flow-chart of the patients included and excluded from the study.

From the Pharmacy database of our institution, we identified 142 patients who received anti-CD20 maintenance from July-2003 to May-2022. 118 patients fulfilled inclusion criteria and signed the informed consent. In **Figure 1** we depict a flow-chart detailing the patients included in the study. Of note, 24 patients were excluded for the following reasons: 21 (87%) had not been vaccinated by August 2022, 2 (8%) had not received frontline anti-CD20 maintenance for B-cell lymphoma and 1 (4%) declined to sign the informed consent. Main characteristics of patients are showed in **Table 1**. Briefly, median age was 64 years (22–89), 52% of cases were male, the most frequent diagnosis was follicular lymphoma (51%), followed by diffuse large B-cell lymphoma (17%) and mantle lymphoma (10%), most cases with advanced III-IV AA stage (82%) and 26% with B-symptoms.

**Table 1:** Main characteristics of patients.

Median age (range)	64 (22-89)
Sex (Male/Female) (%)	61 (52%)/57 (48%)
Diagnosis (%): - Follicular lymphoma - DLBCL - Mantle lymphoma - Marginal lymphoma - CLL/SLL - Other	60 (51%) 20 (17%) 12 (10%) 11 (9%) 7 (6%) 8 (7%)
Ann Arbor Stage (%): - I-II - III-IV	21 (18%) 97 (82%)
B-symptoms (%):	31 (26%)
Induction therapy (%): - R-bendamustine - R-CHOP/R-CVP - R monotherapy - R-GemOx - Fludarabine- based - Intensive approaches - Other	54 (46%) 34 (29%) 14 (12%) 9 (8%) 3 (2%) 3 (2%) 1 (1%)
Median induction cycles (range)	6 (1-9)
Anti-CD20 maintenance (%): - 2 years every 2 months - 2 years every 3 months - 3 years every 2 months	110 (93%) 6 (5%) 2 (2%)
Median maintenance cycles (range)	12 (1-18)
Secondary hypogammaglobulinemia (%): - Yes - No	51 (43%) 67 (57%)
Intravenous immunoglobulin administration (%): - Yes - No	30 (25%) 88 (75%)

DLBCL, diffuse large B-cell lymphoma; CLL/SLL, chronic lymphoid leukemia/small lymphocytic lymphoma; R-CHOP, rituximab, cyclophosphamide, doxorrubicin, prednisone; R-CVP, rituximab, cyclophosphamide, prednisone; R-GemOx, rituximab, gemcitabine, oxaliplatin.

Regarding previous therapy, most patients received R-bendamustine (46%) or R-CHOP/R-CVP (29%) as induction regimen. Median number of induction cycles was 6 (1-9). Most patients received the anti-CD20 maintenance therapy in the frontline setting and using a 2-years every 2-months approach (93%). Median maintenance cycles were 12 (1-18). Associated to anti-CD20 maintenance, 51 patients (43%) showed secondary hypogammaglobulinemia.

### 3.2 SARS-CoV-2 vaccine response and severity of COVID-19

**Figure 2:**
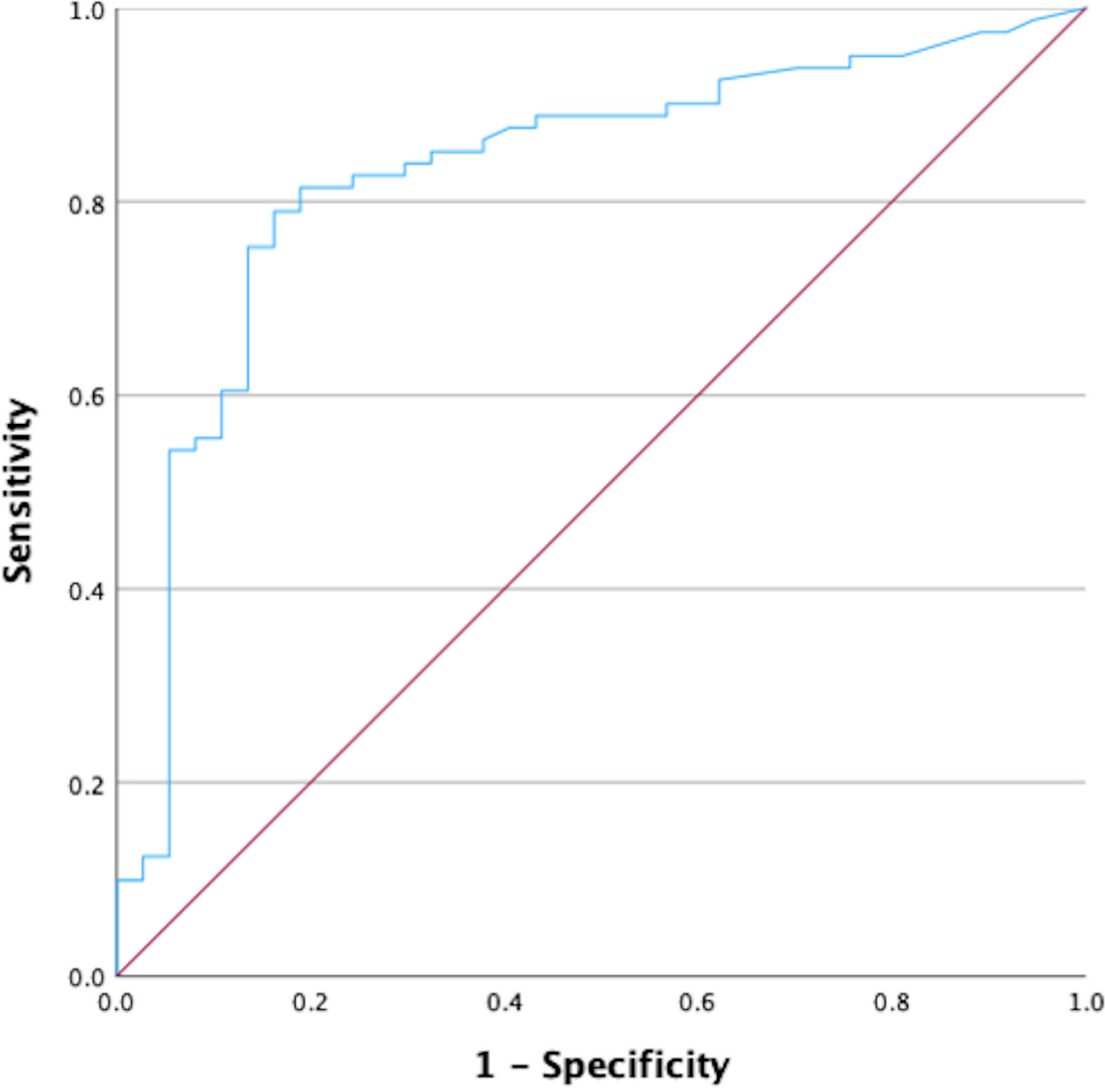
ROC curve to identify the moment of restoration of serological responses to SARS-CoV-2 vaccines after anti-CD20 maintenance.

As shown in **Table 2**, median SARS-CoV-2 vaccine doses received were 3 (1-4), most patients having 3 or 4 doses (83%). Median time since last anti-CD20 dose was 48 months (0-189). Median IgG anti-S quantitative anti-SARS-CoV-2 title was 893.2 AU/mL (0->40000). The rate of vaccination failure of our series was 31%. Median time since last dose of anti-CD20 of patients with vaccination failures was significantly lower (2 months) compared with patients with vaccination success (78 months) (p<0.001). Using ROC curves, we obtained a cutoff for SARS-CoV-2 vaccine serologic response at 24 months from last anti-CD20 dose (area under curve of 0.83; p<0.001) (**Figure 2**). From this cutoff, 90% of patients obtained a successful IgG anti-S level compared to just 36% below that cutoff (p<0.001), which represent a vaccination failure of 63.8%, with no differences between patients vaccinated during anti-CD20 maintenance (63.6%) or during the first 24 months after the last anti-CD20 dose (63.9%). Similarly, there were no differences in the rate of vaccination failure between patients receiving 1-2 vaccine doses and those receiving 3-4 (p=0.6).

**Table 2:** SARS-CoV-2 and COVID-19 infection data.

Median vaccine doses (range)	3 (1-4)	p
Vaccine doses: - 1 - 2 - 3 - 4	2 (2%) 17 (14%) 82 (69%) 17 (14%)	N/A
Median months since last anti-CD20 dose (range)	48 (0-189)	N/A
Median IgG anti-S quantitative anti-SARS-CoV-2 (AU/mL)	893.2 (0->40000)	N/A
Vaccination failure (<260 AU/mL)	37 (31%)	N/A
COVID-19 infection (%)	63 (53%)	N/A
Severity of COVID-19 infection: - Asymptomatic - Mild - Severe - Requiring intensive care	10 (16%) 32 (51%) 15 (24%) 6 (9%)	N/A
Vaccination response according to time from end of anti-CD20 maintenance: - 0-24 months - >24 months	17/47 (36%) 64/71 (90%)	<0.001
Risk of severe COVID-19 according to time from end of anti-CD20 maintenance: - 0-24 months - >24 months	15/29 (52%) 6/34 (18%)	0.007

Considering severity of COVID-19 infection in this series of B-cell lymphomas treated with anti-CD20 maintenance, 63 cases had a COVID-19 infection (53%). From these patients, 33% suffered severe or requiring intensive care COVID-19 while in 67% was mild or asymptomatic. More importantly, the risk of severe COVID-19 was much higher during the first 24 months after last anti-CD20 dose (52%) than after this cutoff (just 18%) (p=0.007). **Table 2** shows main SARS-CoV-2 and COVID-19 characteristics of the series.

### 3.3 Impact of vaccine response, hypogammaglobulinemia and COVID-19 infection on survival

**Figure 3:**
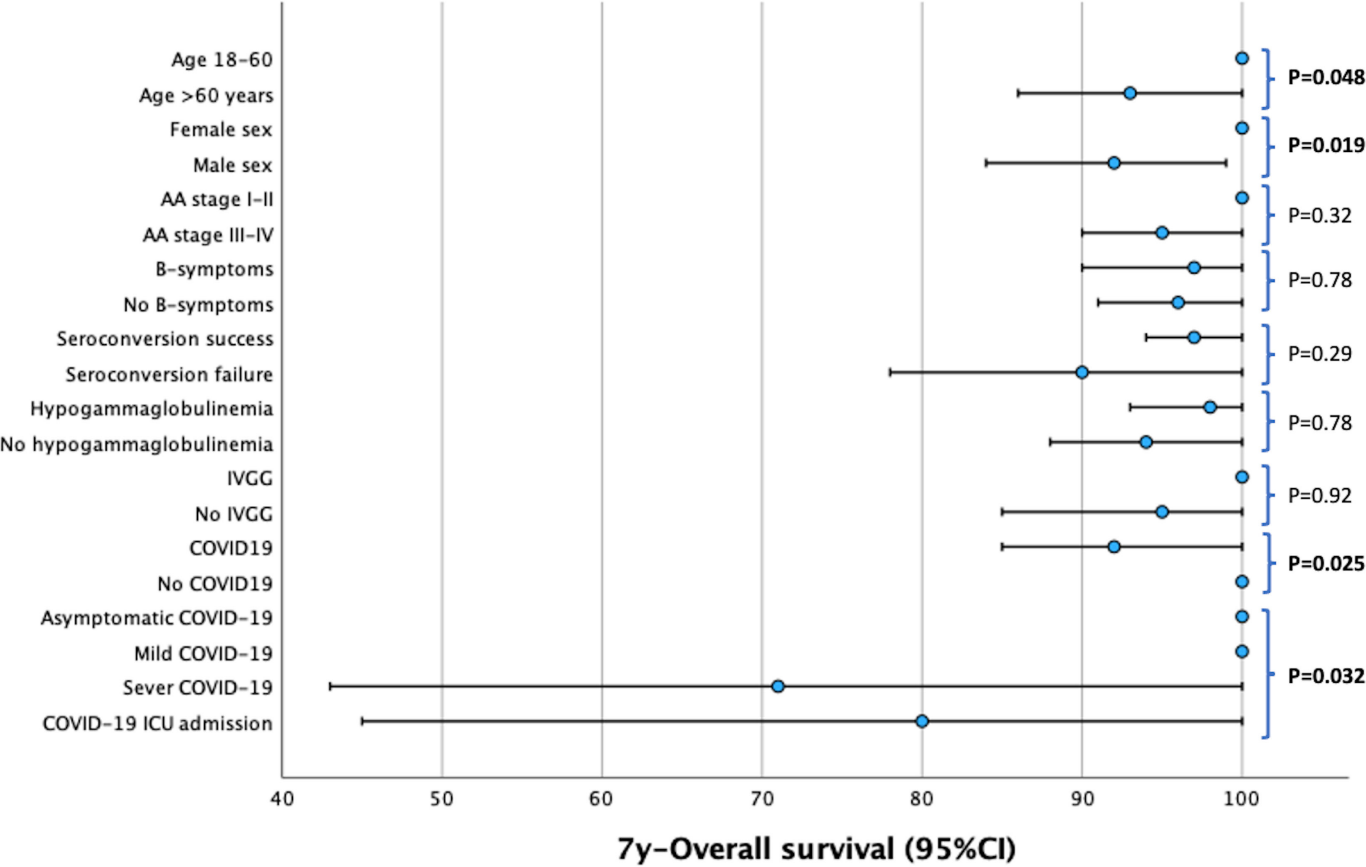
Forest-plot of the univariate analysis of overall survival.

Median follow-up of our series from frontline therapy was 85 months (95%CI: 70-100). 7y-OS was 96% (95%CI: 92-100). Univariate analysis of clinical factors associated with OS is shown in **Table 3**; **Figure 3**. There was no significant impact of vaccine response (p=0.29) or hypogammaglobulinemia (p=0.78). However, the incidence of COVID-19 was associated with a significantly lower OS (p=0.025). Although the difference was only 8% (100% *vs* 92% with COVID19), this difference was related to severe cases (2/15) or ICU cases (1/6), in which the mortality rate associated with COVID-19 was 13% and 17%, respectively. Overall, causes of death were COVID-19 in 3 patients (2.5%) and stroke in 2 cases (1.7%).

**Table 3:** Univariate analysis of clinical factors on overall survival.

	7y-OS (95%CI)	p
Age: - 18-60 - >60	100% (NA) 93% (86-100)	0.048
Sex: - Male - Female	92% (84-99) 100% (NA)	0.019
AA stage: - I-II - III-IV	100% (NA) 95% (90-100)	0.32
B-symptoms: - Yes - No	97% (90-100) 96% (91-100)	0.78
Diagnosis: - Follicular lymphoma - Non-follicular indolent - Mantle-cell lymphoma - DLBCL	94% (88-100) 100% (NA) 100% (NA) 93% (79-100)	0.64
Induction therapy: - Benda-based - CHOP-like - Rituximab monotherapy - Other	93% (86-100) 97% (90-100) 100% (NA) 100% (NA)	0.36
Seroconversion after vaccine: - Success - Failure	97% (94-100) 90% (78-100)	0.29
Hypogammaglobulinemia: - Yes - No	98% (93-100) 94% (88-100)	0.78
Intravenous immunoglobulins (if hypogammaglobulinemia): - Yes - No	100% (NA) 95% (85-100)	0.92
COVID19: - Yes - No	92% (85-100) 100% (NA)	0.025
Severity of COVID-19 infection: - Asymptomatic - Mild - Severe - Requiring intensive care	100% (NA) 100% (NA) 71% (43-100) 80% (45-100)	0.032

NA, not available.

In this series, despite potential B-cell immunosuppression and high rates of B-cell ablation, the COVID-19-related mortality rate was 2.5% in 118 cases. Although this is significant, it appears modest. However, it is important to note that all patients were vaccinated, and a majority of those with hypogammaglobulinemia received intravenous immunoglobulin therapy (59%).

When we evaluated other immunosuppressive factors such as the type of induction therapy, we observed a significantly higher incidence of COVID-19 among patients administered induction therapy with BR at 65%, compared to those treated with R-CHOP/R-CVP at 35% (p=0.036). However, we should note that BR has been the preferred therapy for the most recent patients. Consequently, the median time from the last anti-CD20 dose was 37 months for BR patients and 82 months for R-CHOP/R-CVP patients (p=0.004). Importantly, this higher incidence of COVID-19 did not translate into a significant difference in 7-year overall survival (7y-OS) (93% *vs*. 97%; p=0.36) or in COVID-19-specific death rates (3.7% *vs*. 2.9%; p=0.74).

## 4 Discussion

We present the first data set about the impact on SARS-CoV-2 vaccines efficacy of a particular approach in anti-CD20 therapy for B-cell malignancies, the maintenance treatment. Most anti-CD20 maintenance approaches imply a severe long-term (2-3 years) B-cell ablation. This fact has generated important concerns about their safety in the context of the SARS-CoV-2 pandemic to both physicians and patients. Such concerns may lead to preclude the use of these anti-CD20 maintenance, which consequently may imply a worse control of the B-cell malignancy.

Anti-CD20 therapy efficiently depletes peripheral B-cells that represent only 2% of the total B-cell population. Similarly, there is an impact on peripheral lymphoid tissues but lower on long-lived plasma cells, which do not express the anti-CD20 antigen (12–15). After short-term anti-CD20 induction schemes, such as R-CHOP-like regimens or rituximab monotherapy, the peripheral blood B-cell compartment has been described to recover within 6-9 months after the last anti-CD20 dose (8, 12). However, there is less information regarding long-term anti-CD20 approaches such as anti-CD20 maintenance, but this data could be obtained evaluating a surrogate biomarker of proper B-cell function such as seroconversion after vaccination. To identify the point in which there is a significant change in the ability of seroconvert after vaccination, we evaluated only patients who receiving, or had previously received, anti-CD20 maintenance as part of their frontline therapy. We excluded those who received anti-CD20 maintenance in the second or subsequent treatment lines (**Figure 1**).

In our study, to our knowledge also for the first time, we used ROC curves to calculate the length of main impairment of seroconversion after vaccination in anti-CD20 maintenance approaches: 24 months since the last anti-CD20 dose. The median rate of vaccination failure was 31% in our series, being even higher (64%) during maintenance and up to the first 24 months after the last dose of anti-CD20 maintenance. Beyond this moment, the seroconversion rate improved until 90% (10% vaccination failure rate), showing a much longer impairment on B-cell function after anti-CD20 maintenance therapy compared to short induction regimens.

A recent metanalysis in patients mostly receiving short courses of anti-CD20 therapy, reported even lower seroconversion rates for 2 doses of the pandemic influenza vaccine in patients on active anti-CD20 therapy (12%) and that apparently improved with the time since the last anti-CD20 dose. When comparing patients on active anti-CD20 therapy with controls, the differences in seroconversion rates were less pronounced by an average of 6 to 12 months from last anti-CD20 dose and were similar beyond 12 months (16). To overcome this prologued time to seroconversion it has been proposed to delay anti-CD20 therapy until after vaccination (17). Another recent study proposed that the optimal interval for SARS-CoV-2 vaccination after the final dose of anti-CD20 is 5.5 months, but mostly in patients receiving short courses of anti-CD20 therapy (18).

In the context of our study on SARS-CoV-2 serologic assessment, the high sensitivity and specificity of the CLIA method is especially crucial, ensuring that the antibody responses of individuals, even if weak, are accurately captured. This ensures the validity and robustness of our findings, particularly when drawing conclusions about the impact of treatments or interventions on antibody production and response (19).

The other interesting contribution that we can extract form our series is that anti-CD20 maintenance approaches are safe even in patients with a high degree of humoral immunodeficiency during especially risky situations such as the recent SARS-CoV-2 pandemic. In our patients we had no significant impact on survival of seroconversion failure after SARS-CoV-2 vaccination or hypogammaglobulinemia. However, 25% of these patients received intravenous immunoglobulins therapy, mainly those having symptomatic hypogammaglobulinemia (59%). Patients receiving anti-CD20 maintenance approaches have an increased risk of hypogammaglobulinemia, some of them associated with recurrent infections (20). Some guidelines recommend administering intravenous immunoglobulins to patients with 2 or more non-neutropenic infections in a 6-month period of time (21) and this is also our standard approach. Furthermore, in our series all patients were vaccinated. It is well described that even patients who do not respond to the SARS-CoV-2 vaccines, may develop some degree of T-cell sensibilization that could in part protect or reduce the severity of COVID19 (22).

Another aspect warranting discussion involves the controversial impact of induction immunochemotherapy based on bendamustine (BR) compared to alternatives such as R-CHOP/R-CVP or other options. In our study, we noted a higher incidence of COVID-19 among patients treated with BR, potentially attributable to a higher immunosuppressive activity of bendamustine but also to its status as the preferred therapy for the most recent indolent lymphoma cases, and the corresponding shorter interval since the last anti-CD20 dose. However, it is pivotal to highlight, as previously mentioned, that this increased incidence of COVID-19 did not correlate with a higher incidence of severe COVID-19, shorter OS or higher COVID-19-specific death rates.

Like all retrospective studies, our work is subject to potential bias. Furthermore, we included only those patients who were alive in May 2022, a time when less aggressive SARS-CoV-2 variants were in circulation. Although the impact of these variants might be partially compensated by the less stringent lockdown measures in place, they could have influenced the OS analysis. However, these factors would not affect the seroconversion rates.

Although in our series, vaccination failure or hypogammaglobulinemia did not impact outcomes, COVID19 still had a small but significant effect on mortality (2.5%) and OS. While this percentage is low when looking at the entire series, it is higher for cases that were severe or required ICU admission (13% and 17%, respectively). However, these figures do not significantly differ from the rates reported for the general immunocompetent population (23, 24). They still represent acceptable mortality rates considering the potential B-cell immunosuppression in this group of patients. Given this, we can hypothesize that with the above-mentioned prophylactic measures and close monitoring, there is no justification to broadly preclude the use of anti-CD20 maintenance to any of the well-demonstrated clinical settings in which these approaches have shown important benefit in terms of progression-free survival or even lymphoma cure. Additionally, during the COVID-19 pandemic several anti-SARS-CoV-2 monoclonal antibodies, such as cilgavimab/tixagevimab (25) or sotrovimab (26) have been developed that could help to compensate anti-CD20 maintenance-associated humoral immunodeficiency.

We conclude that anti-CD20 maintenance therapy impairs serological responses to SARS-CoV-2 vaccines. To our knowledge we report for the first time that patients during maintenance and up to 24 months after finishing the last anti-CD20 dose are at a higher-risk of vaccine failure and more severe cases of COVID-19. However, a close monitoring, intravenous immunoglobulin supplementation, if necessary, proper vaccination if available or the use of specific monoclonal antibodies in the case of COVID-19 infection, may overcome the impact on survival of this lack of serological response in high-risk population. In other words, with these measures, anti-CD20 maintenance is a safe procedure that should not be avoided or discontinued even in the case of hypogammaglobulinemia.

## Data availability statement

The raw data supporting the conclusions of this article will be made available by the authors, without undue reservation.

## Ethics statement

The studies involving humans were approved by Balearic Islands ethic committee. The studies were conducted in accordance with the local legislation and institutional requirements. The participants provided their written informed consent to participate in this study.

## Author contributions

AG: Conceptualization, Formal Analysis, Investigation, Methodology, Supervision, Validation, Writing – original draft, Writing – review & editing. AA: Conceptualization, Data curation, Formal Analysis, Investigation, Methodology, Validation, Writing – original draft, Writing – review & editing. MG-R: Conceptualization, Formal Analysis, Investigation, Validation, Writing – original draft, Writing – review & editing. SP: Data curation, Resources, Validation, Writing – review & editing. LG-M: Data curation, Resources, Validation, Writing – review & editing. JM-S: Data curation, Investigation, Resources, Validation, Writing – review & editing. TR: Data curation, Investigation, Resources, Validation, Writing – review & editing. MG-G: Data curation, Investigation, Methodology, Supervision, Validation, Writing – review & editing. JF: Conceptualization, Data curation, Methodology, Supervision, Writing – review & editing. OV: Data curation, Methodology, Supervision, Validation, Writing – review & editing. RA: Data curation, Methodology, Validation, Writing – review & editing. ASl: Conceptualization, Methodology, Supervision, Validation, Writing – review & editing. ASm: Data curation, Resources, Validation, Writing – review & editing. LB: Data curation, Resources, Validation, Writing – review & editing.
